# Provision of mechanical ventilation to pregnant/postpartum women with H1N1 influenza: a case-control study

**DOI:** 10.1186/cc12055

**Published:** 2013-03-19

**Authors:** WE Pollock, R Bellomo, S Webb, I Seppelt, A Davies, E Sullivan, S Morrison, B Howe

**Affiliations:** 1 Mercy Hospital for Women, Heidelburg, Australia; 2 Austin Health, Heidelberg, Australia; 3 Royal Perth Hospital, Perth, Australia; 4 Nepean Hospital, Penrith, Australia; 5 Alfred Hospital, Prahran, Australia; 6 University of New South Wales, Randwick, Australia; 7 Monash University, Prahran, Australia

## Introduction

During the influenza pandemic of 2009, clinicians delivered mechanical ventilation to pregnant women with little evidence to guide practice. The objective of this study was to compare the provision of mechanical ventilation to pregnant/postpartum women and a nonpregnant matched control group admitted to the ICU with H1N1 influenza.

## Methods

A case-control study was conducted following ethics approval. A case was defined as a ventilated pregnant/postpartum woman reported to the Australian and New Zealand INFINITE H1N1 09 study from 1 June 2009 to 31 August 2009. Controls were ventilated nonpregnant women (15 to 49 years) reported to the INFINITE H1N1 09 study during the same time frame. Data were entered into SPSS and analysed using nonparametric statistics; two-tailed *P <*0.05 was considered significant.

## Results

We studied 36 index cases and 38 controls. Index cases were more likely to have a single arterial blood gas (ABG) taken prior to intubation (*P <*0.05). Similar reasons were given for the trigger to intubate (high respiratory rate, low PaO_2_, increased work of breathing) apart from a high PaCO_2_, which was a trigger in the control group only (*P <*0.05). There were no differences in the pre-intubation and post intubation ABGs apart from a lower PaCO_2 _(*P <*0.05) and lower HCO_3 _(*P <*0.05) in cases, and cases presented with a lower haemoglobin (*P <*0.05). There were six difficult intubations documented with no differences between groups. Initial ventilator settings including mode, tidal volume, minute volume and respiratory rate demonstrated no differences. Both groups showed increases in PaO_2 _and PaCO_2_, and a decrease in pH from the pre-intubation to post-intubation ABGs (*P <*0.05).

## Conclusion

There were physiological differences between the two groups with pregnant/postpartum women showing lower PaCO_2 _and HCO_3_. However, initial ventilator support was not significantly different for pregnant/postpartum women compared with controls.

